# Feiyiliu Mixture sensitizes EGFR^Del19/T790M/C797S^ mutant non-small cell lung cancer to osimertinib by attenuating the PRC1/Wnt/EGFR pathway

**DOI:** 10.3389/fphar.2023.1093017

**Published:** 2023-01-19

**Authors:** Jingjing Shi, Shaoyu Hao, Xiantao Liu, Yingying Li, Xin Zheng

**Affiliations:** ^1^ College of First Clinical Medical, Shandong University of Traditional Chinese Medicine, Jinan, China; ^2^ Qingdao Hospital of Traditional Chinese Medicine (Qingdao Hiser Hospital), Qingdao, China; ^3^ Department of Thoracic Surgery, Shandong Cancer Hospital and Institute, Shandong First Medical University and Shandong Academy of Medical Sciences, Jinan, China; ^4^ Department of Respiratory Medicine, Affiliated Hospital of Shandong University of Traditional Chinese Medicine, Jinan, China

**Keywords:** Feiyiliu Mixture, osimertinib, acquired resistance, PRC1/Wnt/EGFR pathway, non-small cell lung cancer

## Abstract

**Introduction:** Osimertinib is a potent epidermal growth factor receptor tyrosine kinase inhibitor (EGFR-TKI) for the treatment of patients with EGFR-mutant non-small cell lung cancer (NSCLC). However, the emergence of acquired resistance due to the EGFR-Del19/T790M/C797S mutation limits the clinical application of osimertinib. Feiyiliu Mixture (FYLM), a clinical experience formula of Chinese medicine, was used to treat lung cancer with good clinical efficacy. In this study, we aimed to investigate the mechanism by which Feiyiliu Mixture delays osimertinib resistance in EGFR-mutant cell lines and EGFR-mutant cell tumor-bearing mice.

**Methods:** The osimertinib-resistant cell models were established in mouse Lewis lung carcinoma (LLC) cells transfected with EGFR-Del19/T790M/C797S mutant lentivirus. In cell experiments, after 48 h of treatment with Feiyiliu Mixture-containing serum, MTT assay was used to detect the relative cell viability, and western blotting was used to detect EGFR protein phosphorylation expression. In animal experiments, C57BL/6J mice were subcutaneously injected with Lewis lung carcinoma cells stably expressing EGFR-Del19/T790M/C797S mutations to construct a xenograft model. After 2 weeks of Feiyiliu Mixture and/or osimertinib treatment, the expression of proliferation-related, apoptosis-related and PRC1/Wnt/EGFR pathway markers was detected by real-time qPCR, western blotting and immunohistochemistry.

**Results:** The results showed that when combined with osimertinib, Feiyiliu Mixture synergistically reduces proliferation and increases apoptosis to improve drug resistance. *In vitro*, Feiyiliu Mixture-containing serum reduced the EGFR phosphorylation. *In vivo*, Feiyiliu Mixture downregulated the expression of cyclin B1 and Bcl-2 while upregulating the level of cleaved Caspase-3 protein, indicating that Feiyiliu Mixture promotes apoptosis. Furthermore, Feiyiliu Mixture reduced the expression of p-EGFR, p-Akt, PRC1 and Wnt pathway-related proteins such as β-catenin, c-Myc and c-Jun.

**Conclusion:** The present study identified that Feiyiliu Mixture inhibited PRC1/Wnt/EGFR pathway activation, reduced proliferation, and promoted apoptosis, thereby increasing the sensitivity of EGFR-mutant non-small cell lung cancer to osimertinib. Our study provided a new idea for Chinese medicine to play a role in enhancing efficacy and reducing toxicity in the treatment of non-small cell lung cancer.

## 1 Introduction

Lung cancer is the most common malignancy and the leading cause of cancer-related deaths, causing a huge social burden in the world (Sung et al., 2021; Siegel et al., 2022). In 2020, there are about 2.2 million new cancer cases (11.4%) and 1.8 million cancer deaths (18%) of lung cancer worldwide (Sung et al., 2021). Non-small cell lung cancer (NSCLC) made up about 85 percent of all lung cancer cases (Molina et al., 2008; Testa et al., 2018). Among them, lung adenocarcinoma is the main histological phenotype of NSCLC, accounting for approximately 55%. Patients with lung cancer are not easily detected at an early stage, and the majority of them are at an advanced stage by the time of clinical diagnosis, losing the opportunity for surgical resection. Current treatments for advanced NSCLC include cytotoxic chemotherapy, radiotherapy, targeted therapy, immunotherapy, and various combination therapies (Miller and Hanna, 2021; Gesthalter et al., 2022; Higgins et al., 2022). With the development of molecular biology research, targeted therapy guided by oncogenic drivers is considered to be an effective means to improve the overall survival and prolong progression-free survival of patients with lung adenocarcinoma (Ramalingam et al., 2020; Ettinger et al., 2022). Epidermal growth factor receptor (EGFR) is considered to be one of the most common driver oncogenes in NSCLC (Harrison et al., 2020; Russo et al., 2020; Cooper et al., 2022). Approximately 50% of Asian patients with lung adenocarcinoma have EGFR-activating mutations, mainly including exon 19 base deletions (Del19) and point mutation in exon 21 (L858R) (Sharma et al., 2007). Moreover, EGFR gene mutation is a biomarker for predicting the effectiveness of targeted therapy. Therefore, genetically mutated EGFR is an important target for targeted therapy in lung adenocarcinoma.

According to the National Comprehensive Cancer Network (NCCN) guidelines for NSCLC (Ettinger et al., 2022), tyrosine kinase inhibitors (TKIs) are recommended as first-line therapy for patients with EGFR-mutant advanced NSCLC. The first and second-generation EGFR-TKIs (gefitinib, erlotinib and afatinib) targeting EGFR tyrosine kinase domain achieved marked clinical efficacy, but unfortunately, acquired resistance occurs 9–14 months later (Park et al., 2016; Hsu et al., 2018). The mechanisms of acquired resistance to EGFR-TKIs involve the second-site mutations of EGFR (such as T790M gatekeeper mutation), activation of the bypass signaling pathways, epithelial-mesenchymal transition (EMT), the transformation of NSCLC to small cell lung cancer tissue, etc. (Westover et al., 2018; Wu and Shih, 2018). Among them, T790M mutation occurs in at least 50% of the patients (Remon et al., 2018). Osimertinib, a third-generation irreversible EGFR-TKI, selectivity targeted against both activating mutations and T790M resistance mutations. However, after 9–13 months of treatment, there was only a transient benefit followed by osimertinib resistance due to the C797S mutation (Thress et al., 2015; Du et al., 2021). Currently, there is no effective therapeutic strategy to overcome the triple mutation (Del19/T790M/C797S)-mediated drug resistance problem. Clearly, exploring the resistance mechanism and searching for new therapeutic targets to delay and reverse EGFR-TKIs resistance in lung adenocarcinoma is an urgent problem to be solved in tumor-targeted therapy and it is also essential for the treatment of NSCLC.

Research has shown that the protein regulator of cytokinesis 1 (PRC1) is associated with the mitotic process of tumor cells and is highly expressed in various carcinomas (Li et al., 2018). As well as, PRC1 promotes lung adenocarcinoma cells proliferation, metastasis and tumorigenesis by activating the Wnt/β-catenin signaling pathway (Zhan et al., 2017). Besides, the activation of EGFR and its downstream signaling pathways by the Wnt/β-catenin pathway is an important molecular mechanism for the development of EGFR-TKI resistance (Arasada et al., 2018). Hence, inhibition of the PRC1/β-catenin/EGFR pathway may be an important molecular pathway for overcoming EGFR-TKI resistance.

Traditional Chinese Medicine (TCM) has certain advantages in the treatment of chronic diseases and diseases with complex factors, especially in anti-tumor (Shao et al., 2021). In addition, TCM and molecular targeted drugs have synergistic anti-cancer effects on lung cancer patients with EGFR-TKI resistance (Li et al., 2019; Wang et al., 2021b). Feiyiliu Mixture (FYLM), a clinical experience formula of Chinese medicine, is composed of Huangqi, Banzhilian, Baizhu, Baihuasheshecao, Renshen, Fuling, Zhebeimu, Shancigu, Yiyiren and Gancao. Our previous studies have demonstrated that compared with erlotinib alone, FYLM combined with erlotinib not only inhibits tumor growth and improves symptoms but also effectively alleviates side effects in patients with advanced lung adenocarcinoma (Li, 2010). In addition, FYLM can reduce the mRNA expression of EGFR and regulate the PI3K/Akt pathway downstream of EGFR (Cao et al., 2016; Peng et al., 2019). Therefore, we speculated that FYLM may be involved in the process of EGFR-TKI resistance and may complement targeted drugs. However, the underlying molecular mechanism remains unknown.

In the present study, we aimed to investigate whether FYLM could delay osimertinib resistance by regulating the PRC1/Wnt/EGFR pathway in EGFR triple-mutant LLC cells *in vitro* and an EGFR triple-mutant xenograft mouse model *in vivo*.

## 2 Materials and methods

### 2.1 Preparation of FYLM decoction

FYLM consists of ten Chinese herbal medicines, including 18 g of Huangqi [*Astragalus membranaceus* (Fisch.) Bge.], 24 g of Banzhilian (*Scutellaria barbata* D. Don), 12 g of Baizhu (*Atractylodes macrocephala* Koidz.), 20 g of Baihuasheshecao [*Oldenlandia diffusa* (Willd.) Roxb.], 9 g of Renshen (*Panax ginseng* C. A. Mey.), 15 g of Fuling [*Poria cocos* (Schw.) Wolf], 15 g of Zhebeimu (*Fritillaria thunbergii* Miq.), 20 g of Shancigu [*Cremastra appendiculata* (D. Don) Makino], 20 g of Yiyiren [*Coix lacryma*—*jobi L. var. ma*—*yuen* (Roman.) Stapf] and 6 g of Gancao (*Glycyrrhiza uralensis* Fisch.). All the Chinese medicine pieces were provided by Bozhou Huqiao Pharmaceutical Co., Ltd (Bozhou, China) and authenticated by Prof. Feng Li of Shandong University of Traditional Chinese Medicine. For *in vivo* animal studies, all herbs (159 g in total) were soaked in ten times cold distilled water for 1 h and then decocted for 40 min. After filtering the solution, the dregs of decoction were decocted again in eight times distilled water for 30 min. After combining two filtrates, the FYLM decoction was concentrated to 66 ml under a rotary evaporator (N-1300, Shanghai Ailang Instrument Co., Ltd, Shanghai, China). At last, 2.4 g (crude drug)/ml FYLM decoction was prepared and stored at −20°C.

### 2.2 UPLC-Q-Orbitrap-MS analysis

The composition analysis of FYLM decoction was detected using a chromatograph (instrument model: UltiMate 3000 RS) and a Q Exactive high-resolution mass spectrometer [Thermo Fisher Scientific (China) Co. Ltd.]. 1 ml of 80% methanol was mixed with 200 µl of FYLM solution and vortexed for 10 min. After centrifugation at 20,000 g for 10 min at 4°C, the supernatant was filtered for subsequent studies. Chromatographic analysis was performed on an AQ-C18 column (150 mm × 2.1 mm, 1.8 µm, Welch) at 35°C with an injection volume of 5 µl. The chromatographic gradient elution procedure: 98% aqueous phase, 2% organic phase at 1 min; 80% aqueous phase, 20% organic phase at 5 min; 50% aqueous phase, 50% organic phase at 10 min; 20% aqueous phase, 80% organic phase at 15 min; 5% aqueous phase, 95% organic phase at 20 min; 5% aqueous phase, 95% organic phase at 27 min; 98% aqueous phase, 2% organic phase at 28 min; 98% aqueous phase, 2% organic phase at 30 min. The mass spectrometry analysis was performed using a full mass/dd-MS2 detection method with a positive and negative ion switching scan. Data collected from the high-resolution liquid mass were initially analyzed by CD2.1 (Thermo Fisher) and then compared to a database (mzCloud).

### 2.3 Preparation of FYLM-containing serum

Twenty male Sprague-Dawley rats (weighting 180 ± 10 g) were purchased from Vital River Laboratory Animal Technology Co., Ltd. (Beijing, China) [animal License number: SCXK (Jing) 2016-0006]. Animals were housed in a 12/12 h light/dark cycle at 23°C ± 2°C with 50%–60% relative humidity and free of the specific pathogens [Laboratory use of animal license number: SYXK(Lu)2017-0022], with free access to food and water (Geng et al., 2021).

After 1 week of adaptive feeding, the animals were randomly divided into two groups, the control group and the FYLM administration group. The clinical dose of FYLM in adults is 159 g/60 kg and the gavage dose for rats is 6.3 times the clinical dose based on body surface area. The rats of FYLM administration group were given FYLM at the dose of 16.7 g/kg by gavage. While rats in the control group were given saline gavage (1.5 ml/100 g body weight). After 6 days of continuous gavage, rats were anesthetized with 3% pentobarbital sodium (45 mg/kg, intraperitoneal injection) (Sun et al., 2004), and blood was collected from the abdominal aorta. After resting for 1 h at room temperature, the serum was obtained by centrifugation at 3,000 rpm for 15 min at 4°C. Subsequently, it was inactivated in a water bath at 57°C for 30 min. After that, the bacteria were removed by filtration through a 0.22 µm microporous membrane and stored in a refrigerator at −80°C for *in vitro* experiments.

### 2.4 Cell culture

Mouse Lewis lung carcinoma (LLC) cell line and human NSCLC cell line (H1975) were purchased from the Cell bank, Type Culture Collection of Chinese Academy of Sciences (Shanghai, China). LLC cells were maintained in Dulbecco’s Modified Eagle Medium (DMEM, Gibco, Beijing, China) containing 10% fetal bovine serum (FBS, ExCell Bio, Shanghai, China) and 1% Penicillin Streptomycin solution (Yu et al., 2020a). H1975 cells were cultured in RPMI 1640 Medium (Invitrogen, 11875-093) with 10% FBS, 1% Glutamax (Invitrogen, 35050061), and 1% Sodium Pyruvate 100 mM Solution (Invitrogen, 11360070) (Jiang et al., 2021). All cells were cultured in a humidified incubator at 37°C and 5% CO_2_.

### 2.5 Lentivirus transfection of LLC cells

EGFR triple-mutant cells were generated by transfection with lentiviruses harboring the gene sequence encoding EGFR^Del19/T790M/C797S^ mutations in LLC cells. Construction of the overexpression lentiviral vector and concentration of viruses was performed by Shanghai Genechem Co., Ltd. (Shanghai, China). Based on the results of the pre-experiment, the optimal multiplicity of infection (MOI) for EGFR^Del19/T790M/C797S^ overexpression and empty vector lentivirus infection of LLC cells was 100. Before infection, LLC cells were plated in 6-well plates at 3 × 10^5^ cells per well overnight, and then the supernatant was replaced with 1 ml complete medium supplemented with lentivirus. After 14 h of infection, the lentivirus-containing medium was discarded and replaced with 2 ml fresh complete medium. After 72 h of infection, the stably transfected cell lines were selected by 2 μg/ml puromycin. The transfection efficiency was assessed by observing fluorescence intensity, and the EGFR^Del19/T790M/C797S^ overexpression levels were identified by real-time qPCR and western blotting.

### 2.6 Cell viability assays

LLC triple-mutant cells and H1975 cells were seeded at 1 × 10^4^ cells per well in 96-well plates for 24 h. Then, cells were treated with different concentrations of FYLM-containing serum. After 48 h of intervention, 100 µl medium containing 20% MTT (5 mg/ml, Solarbio, Beijing, China) was added to each well. After incubation at 37°C for 4 h, 150 µl dimethylsulfoxide (DMSO) was added to each well and shaken on a shaker at low speed for 10 min to dissolve the formazan crystals. In the end, the absorbance values of each well were measured at 490 nm (Lin et al., 2015) using a full-wavelength microplate reader (BioTek, United States).

### 2.7 Real-time quantitative PCR

Total RNA was extracted from cells or tumor tissues by Trizol reagent (Invitrogen, Thermo Fisher Scientific, United States). A reverse transcription reaction was performed using a 5x All-In-One RT MasterMix kit (Abm, Canada) to synthesize cDNA from total RNA. qPCR assays were detected by the SYBR Green PCR Master Mix kit (DBI Bioscience, Germany) in accordance with the manufacturer’s instructions on a real-time fluorescence quantitative PCR instrument (Roche LightCycler 480II, Mannheim, Germany). The relative expression of genes was measured using the 2^−△△CT^ method (Michaelidou et al., 2013), and GAPDH was used as a normalization control. Each sample was tested three times in duplicate. The primer sequences are shown in Table 1.

**TABLE 1:** The primer sequences used for real-time quantitative PCR.

Gene	Sequence (5′ to 3′)	
EGFR	Forward primer	GCGATTCAGCAACAACC
	Reverse primer	CATTGGGACAGCTTGGA
Akt	Forward primer	TAACGGACTTCGGGCTGT
	Reverse primer	TTC​TCG​TGG​TCC​TGG​TTG​T
GAPDH	Forward primer	TGT​TTC​CTC​GTC​CCG​TAG​A
	Reverse primer	ATC​TCC​ACT​TTG​CCA​CTG​C

### 2.8 Western bolting

After treatments, cells or tumor tissues were lysed for 20 min on ice in RIPA lysis buffer (Solarbio, Beijing, China) supplemented with protease inhibitor (PMSF) and phosphatase inhibitor (APExBIO, MA, United States). And then the supernatant-containing proteins were collected by centrifugation at 12,000 rpm for 20 min at 4°C. Protein concentration measurements were performed using a BCA protein assay kit (Beyotime, Shanghai, China). Protein samples of 40 µg were isolated on 8% or 12% SDS-polyacrylamide gels and transferred onto the polyvinylidene fluoride (PVDF) membranes (0.45 µm, Millipore, Billerica, MA, United States). After blocking with 5% skim milk in TBST at room temperature for 2 h, membranes were incubated separately with the specific primary antibodies at 4°C overnight. The primary antibodies were as follows: EGFR (#4267, Cell Signaling Technology, 1:1000), p-EGFR (#3777s, Cell Signaling Technology, 1:1000), Cyclin B1 (#4138, Cell Signaling Technology, 1:1000), Bcl-2 (#3498, Cell Signaling Technology, 1:1000), Cleaved Caspase-3 (#9661, Cell Signaling Technology, 1:1000), PRC1 (BM3910, BOSTER, Wuhan, China, 1:1000), β-catenin (BA0426, BOSTER, Wuhan, China, 1:1000), c-Myc (PB9092, BOSTER, Wuhan, China, 1:1000), c-Jun (#9165, Cell Signaling Technology, 1:1000), Akt (ab179463, Abcam, 1:10000), p-Akt (ab192623, Abcam, 1:1000), β-actin (BA2305, BOSTER, Wuhan, China, 1:5000), GAPDH (A00227-1, BOSTER, Wuhan, China, 1:1000). After washing 3 times with TBST for 5 min each time, the membranes were incubated with secondary antibodies conjugated to horseradish peroxidase (BOSTER, Wuhan, China, 1:5000) for 1 h at room temperature. Finally, the bands were detected by an automated chemiluminescence gel imaging system (GE Amersham Imager600, United States), and the grayscale value was measured by ImageJ software (NIH, Bethesda, MD).

### 2.9 Triple-mutant EGFR xenograft-bearing mouse model

6-week-old male C57BL/6J mice, weighing 18–20 g, were purchased from Jinan Pengyue experimental Animal Breeding Co., Ltd. (Jinan, China) [animal License number: SCXK(Lu)2019-0003]. LLC cells stably transfected with EGFR^Del19/T790M/C797S^-mutant or empty vector lentivirus were harvested and washed 2 times with PBS. Then, 1 × 10^6^ cells were suspended in 100 µl PBS and injected subcutaneously into the right side of the flank region of C57BL/6J mice. The equivalent dose was converted from human and mouse body surface area (9.1 times), and the dose administered to mice was 24 g/kg. Body weight and tumor volume of mice were recorded every 3 days, and tumor volume was represented by 0.5 × length × width^2^ (mm^3^) (Chen et al., 2021). When the tumor volume reached about 200 mm^3^ (7 days after tumor inoculation) (Uchibori et al., 2017), the mice were randomly divided into seven groups (*n* = 5), empty vector control group (0.5% CMC-Na, Selleckchem, Houston, TX, United States, catalog number: S6703), overexpression control group (0.5% CMC-Na, Selleckchem, Houston, TX, United States, catalog number: S6703), 24 g/kg FYLM group, 48 g/kg FYLM group, osimertinib group (50 mg/kg, AZD9291, Selleckchem, Houston, TX, United States, catalog number: S7279), osimertinib +24 g/kg FYLM group (50 mg/kg osimertinib plus 24 g/kg FYLM) and osimertinib +48 g/kg FYLM group (50 mg/kg osimertinib plus 48 g/kg FYLM). After 2 weeks of continuous gavage, the tumor tissue was obtained and stored at −80°C or fixed with paraformaldehyde for further experiments.

### 2.10 H&E staining

The tumor tissues were dehydrated and embedded in paraffin, and then cut into 5 µm sections. Sections were stained with hematoxylin and eosin and then observed using an optical microscope.

### 2.11 Serum analysis

Blood samples were collected from the mouse retro-orbital venous plexus. After resting at room temperature, the blood samples were centrifuged at 1,500 g for 30 min to obtain the supernatant. According to the manufacturer’s instructions, alanine aminotransferase (ALT), aspartate aminotransferase (AST), alkaline phosphatase (ALP), creatinine (CRE), blood urea nitrogen (BUN) levels in serum samples were determined using the kit from Nanjing Jiancheng (Nanjing, China).

### 2.12 Immunohistochemistry staining

The tumor sections were dewaxed, antigen repaired in a microwave oven, and incubated with 3% H_2_O_2_ for 20 min at room temperature. The sections were incubated overnight at 4°C with an anti-Ki67 antibody (GB111499, Servicebio, 1:500) and an anti-β-catenin antibody (GB11015, Servicebio, 1:1000). Finally, the protein expression of Ki67 and β-catenin was observed under the optical microscope.

### 2.13 TUNEL staining

TUNEL staining of tumor sections was performed to detect apoptosis using the TUNEL assay kit (G1507, Servicebio, Wuhan, China) according to the manufacturer’s instructions. The paraffin sections were dewaxed, rehydrated, and incubated with proteinase K working solution for 20 min at 37°C. After incubation with 3% H_2_O_2_ at room temperature for 20 min, the sections were blocked with TUNEL reaction at 37°C for 1 h in a flat wet box. Positive expression was observed under an optical microscope.

### 2.14 Statistical analysis

The data were analyzed using SPSS 23.0 software (SPSS, Inc., Chicago, IL, United States). Mean ± standard deviation (SD) was used to reveal data. One-way ANOVA analysis followed by Fisher’s least-significant difference (LSD) was used to compare multiple-group statistical differences. A value of *p* < 0.05 was considered statistically significant.

## 3 Results

### 3.1 Identification of the chemical composition of FYLM decoction

**FIGURE 1:**
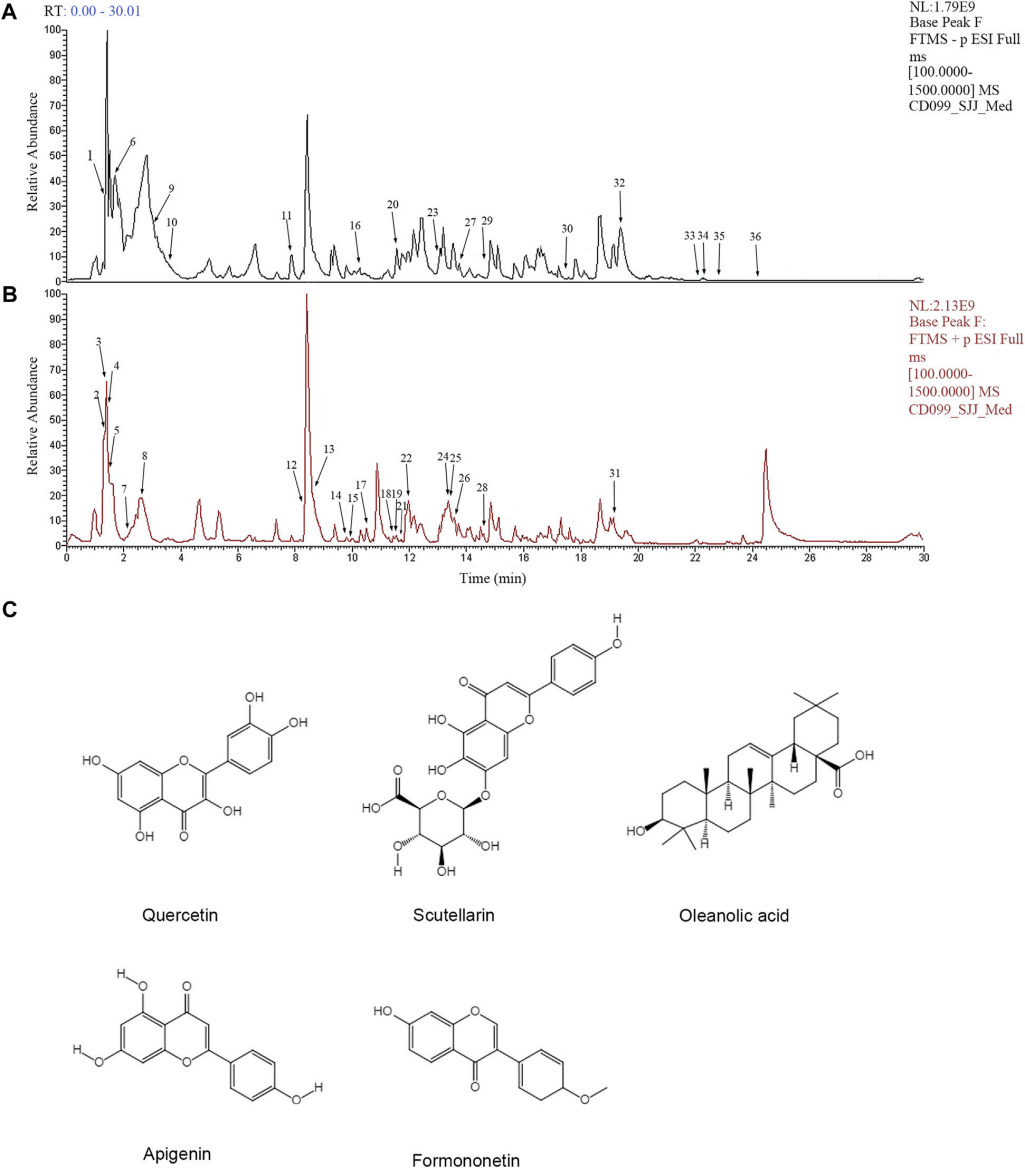
Analysis of the main chemical components of Feiyiliu Mixture (FYLM) by UPLC-Q-Orbitrap-MS in **(A)** negative ion mode and **(B)** positive ion mode. **(C)** Typical chemical structure formulas of components.

To analyze the main components of FYLM, we used combination of Ultra-Performance Liquid Chromatography and Quadrupole-Orbitrap mass spectrometry (UPLC-Q-Orbitrap-MS) method. Chinese medicine samples matched 710 compounds in mzCloud. As shown in Table 2, 36 kinds of compounds were identified by screening with the Traditional Chinese Medicine Systems Pharmacology Database and Analysis Platform (TCMSP, https://old.tcmsp-e.com/tcmsp.php). We found that the main components of huangqi include Choline, Betaine, Nicotinic acid, Coumarin, Chlorogenic acid, Caffeic acid, Ononin, Daidzein, Soyasaponin I, Linoleic acid. The main components of banzhilian include 4-Hydroxybenzaldehyde, Phenylacetaldehyde, Quercetin, Apigenin, Scutellarin, Eriodictyol, Baicalin, Hispidulin. The main components of baizhu include L-Histidine, DL-Arginine, L-Glutamic acid, L-Isoleucine, Uridine, L-Tyrosine. And the main components of baihuasheshecao include Geniposidic acid, 4-Methoxycinnamic acid, Rutin. The total ion chromatograms of FYLM were shown in Figure 1A (negative ion mode) and Figure 1B (positive ion mode). Figure 1C manifested the chemical structure formula corresponding to the typical peaks.

**TABLE 2:** Chemical composition information in FYLM.

NO.	Name	Formula	Reference ion	RT [min]	Calc. MW	mzCloud best match	Corresponding herbs
1	L-Histidine	C6 H9 N3 O2	[M-H]-1	1.293	155.0683	97.8	Baizhu
2	DL-Arginine	C6 H14 N4 O2	[M+H]+1	1.388	174.1114	85.8	Baizhu
3	Choline	C5 H13 N O	[M+H]+1	1.392	103.1001	93.5	Huangqi
4	L-Glutamic acid	C5 H9 N O4	[M+H]+1	1.408	147.0529	95.3	Baizhu
5	Betaine	C5 H11 N O2	[M+H]+1	1.44	117.0791	93.2	Huangqi
6	Isocitric acid	C6 H8 O7	[M-H]-1	1.617	192.026	70.8	Renshen
7	Nicotinic acid	C6 H5 N O2	[M+H]+1	2.131	123.0322	99.8	Huangqi
8	L-Isoleucine	C6 H13 N O2	[M+H]+1	2.602	131.0946	99.8	Baizhu
9	Succinic acid	C4 H6 O4	[M-H]-1	3.116	118.0252	36.1	Shancigu
10	Uridine	C9 H12 N2 O6	[M-H]-1	3.626	244.0693	81.8	Baizhu
11	Geniposidic acid	C16 H22 O10	[M-H]-1	7.838	374.1209	86.5	Baihuasheshecao
12	Dimethyl phthalate	C10 H10 O4	[M+H]+1	8.249	194.0579	60.4	Gancao
13	Coumarin	C9 H6 O2	[M+H]+1	8.675	146.0367	60.1	Huangqi
14	Chlorogenic acid	C16 H18 O9	[M+H]+1	9.804	354.0947	99.7	Huangqi
15	4-Hydroxybenzaldehyde	C7 H6 O2	[M+H]+1	9.817	122.037	97.2	Banzhilian
16	Caffeic acid	C9 H8 O4	[M-H]-1	10.256	180.0416	99.1	Huangqi
17	Vanillin	C8 H8 O3	[M+H]+1	10.656	152.0473	94.4	Yiyiren
18	L-Tyrosine	C9 H11 N O3	[M+H]+1	11.388	181.0739	69.9	Baizhu
19	4-Methoxycinnamic acid	C10 H10 O3	[M+H]+1	11.439	178.0626	66.4	Baihuasheshecao
20	Phenylacetaldehyde	C8 H8 O	[M-H]-1	11.561	120.0561	99.6	Banzhilian
21	Quercetin	C15 H10 O7	[M+H]+1	11.706	302.0422	95.7	Banzhilian
22	Isoliquiritigenin	C15 H12 O4	[M+H]+1	11.96	256.0729	99.3	Gancao
23	Rutin	C27 H30 O16	[M-H]-1	12.965	610.1525	99.7	Baihuasheshecao
24	Apigenin	C15 H10 O5	[M+H]+1	13.291	270.0521	99.3	Banzhilian
25	Scutellarin	C21 H18 O12	[M+H]+1	13.371	462.079	98.2	Banzhilian
26	Ononin	C22 H22 O9	[M+H]+1	13.502	430.1256	98.5	Huangqi
27	Eriodictyol	C15 H12 O6	[M-H]-1	13.699	288.0634	69.9	Banzhilian
28	Baicalin	C21 H18 O11	[M+H]+1	14.594	446.0842	97.1	Banzhilian
29	Formononetin	C16 H12 O4	[M-H]-1	14.732	268.0734	99.5	Gancao
30	Daidzein	C15 H10 O4	[M-H]-1	17.534	254.0576	98.3	Huangqi
31	Soyasaponin I	C48 H78 O18	[M+H]+1	19.215	942.5178	75.5	Huangqi
32	Hispidulin	C16 H12 O6	[M-H]-1	19.444	300.0631	88.9	Banzhilian
33	Oleanolic acid	C30 H48 O3	[M-H]-1	22.062	456.3603	99.2	Gancao
34	Linoleic acid	C18 H32 O2	[M-H]-1	22.291	280.2401	100	Huangqi
35	Palmitic acid	C16 H32 O2	[M-H]-1	22.808	256.2399	100	Fuling
36	Stearic acid	C18 H36 O2	[M-H]-1	24.168	284.2715	99.5	Yiyiren

### 3.2 FYLM-containing serum inhibits the proliferation of EGFR mutant cells and reduces EGFR phosphorylation

**FIGURE 2:**
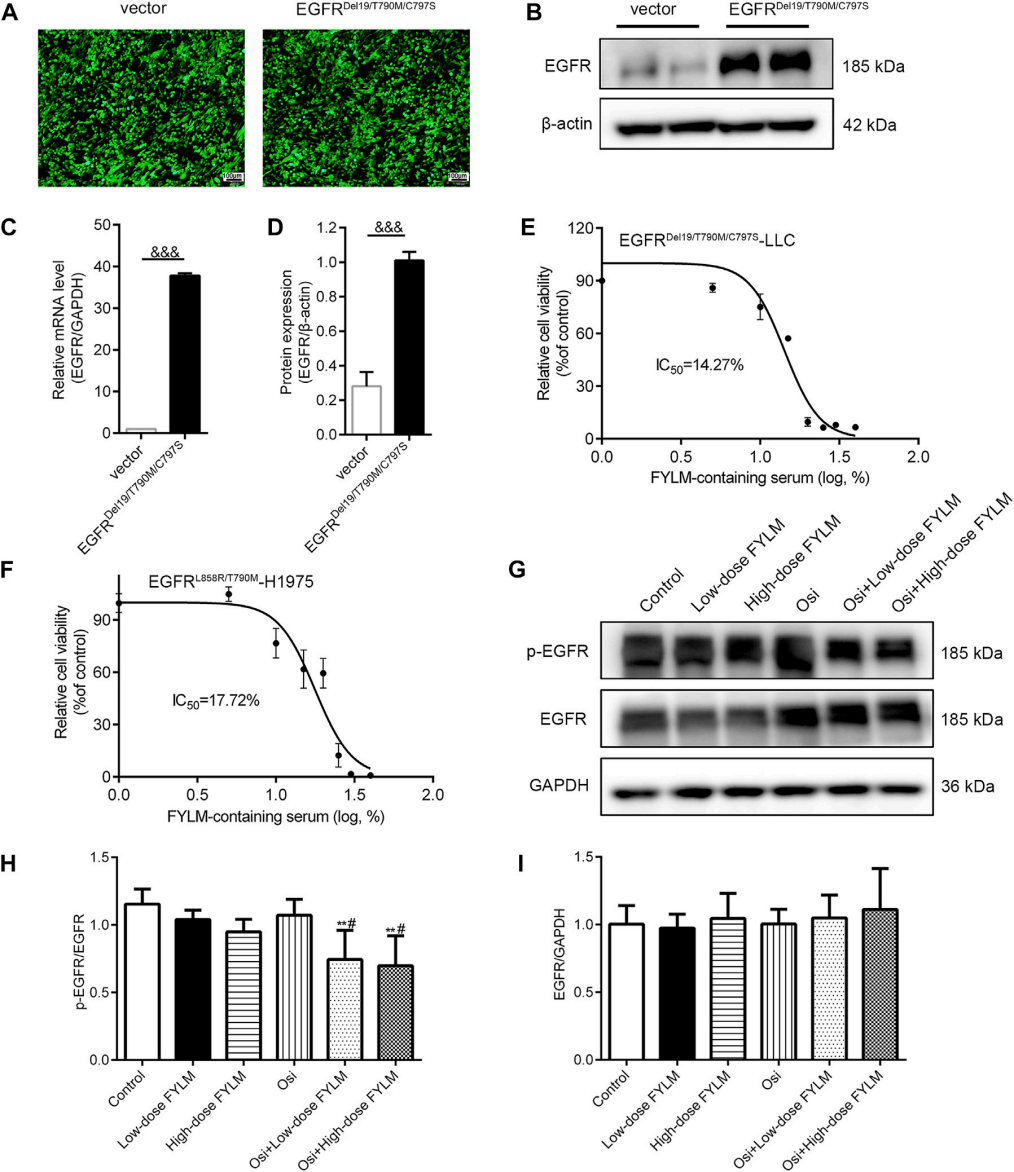
FYLM-containing serum inhibited cell proliferation and reduced EGFR phosphorylation *in vitro*. The **(A)** fluorescence intensity, **(B)** western blot and **(C)** real-time qPCR results were used to identify the transfection effect of the empty vector and EGFR^Del19/T790M/C797S^ mutant lentivirus in LLC cells. **(D)** Quantitative analysis of EGFR protein levels. **(E,F)** MTT assays determined relative cell viability in LLC-EGFR-Del19/T790M/C797S cells and H1975-EGFR-L858R/T790M cells treated with FYLM-containing serum for 48 h **(G)** LLC-EGFR-Del19/T790M/C797S cells were treated with low and high doses of FYLM-containing serum and Osi alone or in combination. p-EGFR and EGFR protein were detected by western blotting. **(H,I)** Quantitative analysis of p-EGFR and EGFR protein levels. ^&&&^ *p* < 0.001 vs. vector; **p < 0.01 vs. Control; ^#^ *p* < 0.05 vs. Osi. Osi, osimertinib. All data are shown as mean ± SD.

To determine whether EGFR^Del19/T790M/C797S^ is stably expressed in LLC cells, we analyzed fluorescence intensity, mRNA and protein levels of EGFR. By fluorescence microscopy, both overexpression and empty vector lentiviruses-transfected LLC cells exhibited enhanced fluorescence signals, indicating normal expression of fluorescent marker genes and successful transfection (Figure 2A). Based on the real-time qPCR results (Figure 2C), the EGFR gene expression in the overexpression group was 37-fold higher than that in the empty vector group. Similarly, the protein levels of EGFR were significantly higher in the overexpression group (Figures 2B, D). The above results suggested that LLC cells were successfully transfected with EGFR^Del19/T790M/C797S^ expressing lentivirus. Next, cell viability was measured by treating with various concentrations of FYLM-containing serum for 48 h in EGFR^Del19/T790M/C797S^-mutated and EGFR^L858R/T790M^-mutated cells. As shown in Figure 2E, FYLM-containing serum dose-dependently inhibited the proliferation of LLC-triple mutant cells (IC_50_ = 14.27%). In addition, FYLM-containing serum can inhibit the proliferation of H1975-L858R/T790M cells (Figure 2F, IC_50_ = 17.72%). In order to further verify the effect of FYLM-containing serum on LLC-triple mutant cells, we detected the protein expression of p-EGFR by western blotting. The results suggested that low and high doses of FYLM-containing serum can reduce EGFR phosphorylation compared with the control group. In addition, the combination of FYLM-containing serum and osimertinib had a more significant inhibitory effect compared with the osimertinib alone group. However, there was no significant differences between high and low doses (Figures 2G–I).

### 3.3 The combination of FYLM and osimertinib inhibits tumor growth in triple-mutant EGFR xenograft mouse model

**FIGURE 3:**
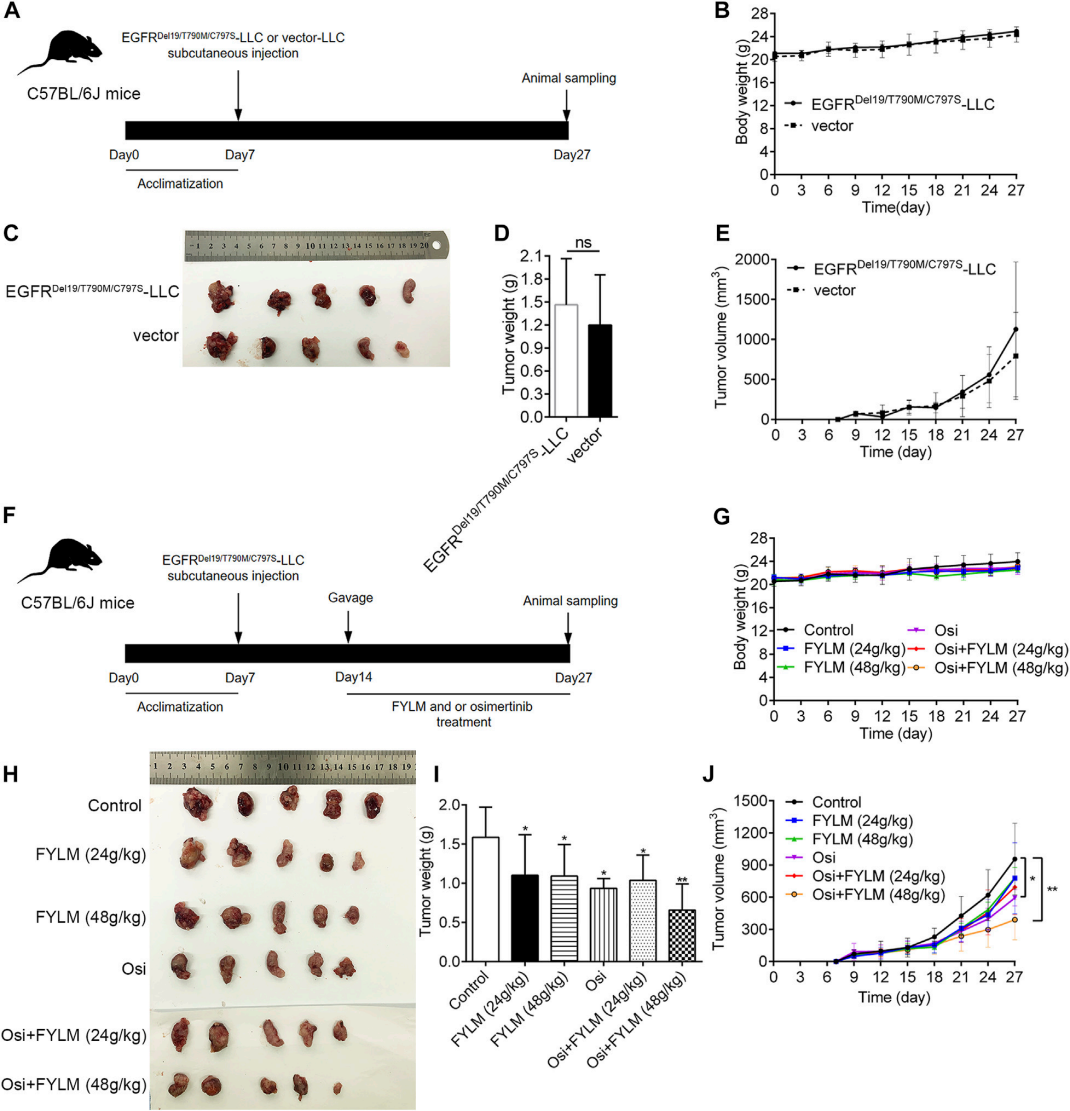
FYLM combined with osimertinib inhibited tumor progression in EGFR-Del19/T790M/C797S LLC tumor-bearing mice *in vivo*. **(A,F)** Flowchart of animal experiments. **(B,G)** Body weights of mice in each group were measured every 3 days. **(C,H)** Photograph of tumor tissues from xenograft mouse. **(D,I)** Tumor weight and **(E,J)** tumor volumes of mice were treated by FYLM and/or osimertinib for 2 weeks. *p < 0.05 and **p < 0.01 vs. Control. Osi, osimertinib. All data are shown as mean ± SD.

**FIGURE 4:**
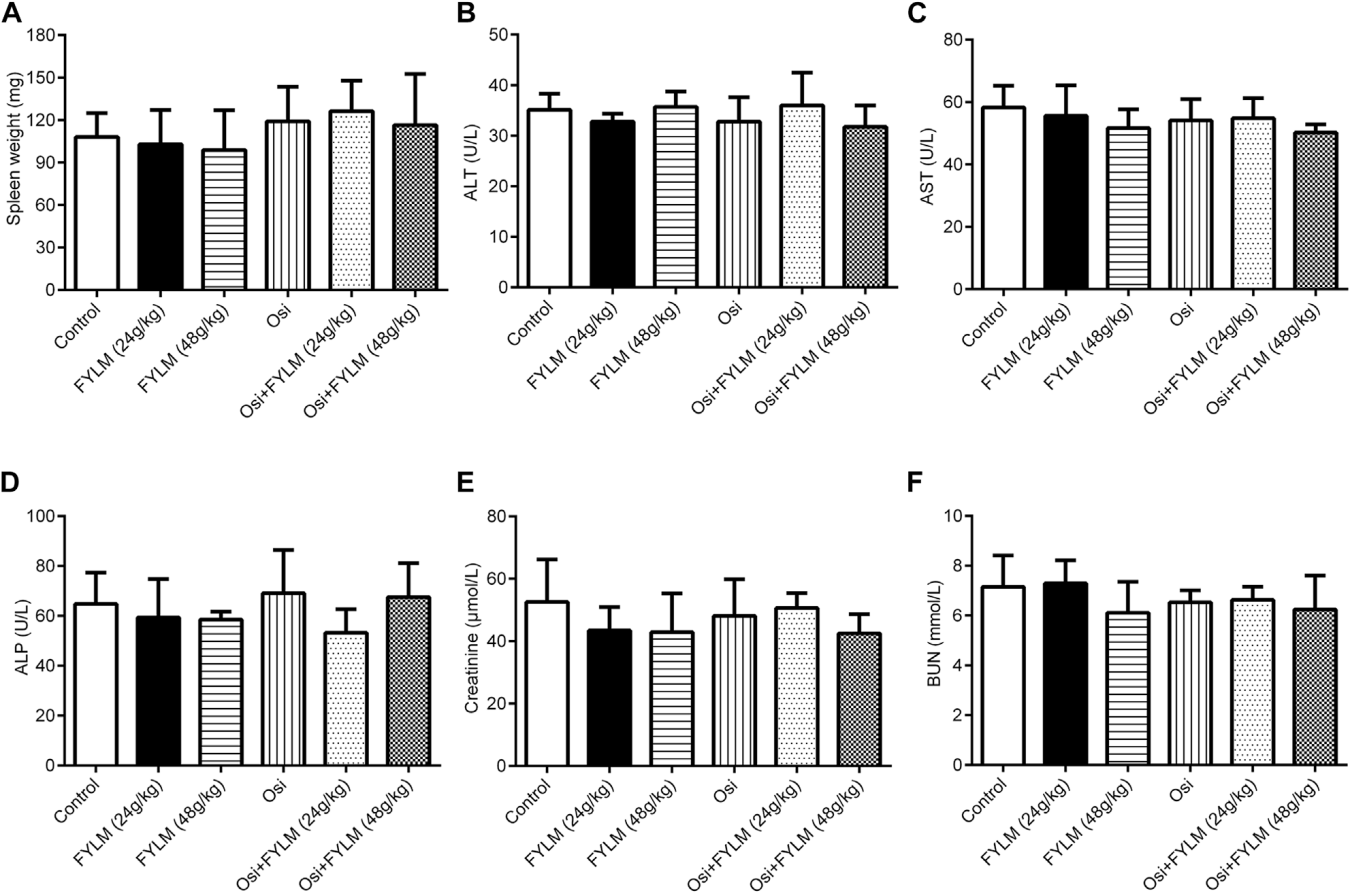
FYLM combined with osimertinib has no obvious toxicity to EGFR-Del19/T790M/C797S LLC tumor-bearing mice. **(A)** Spleen weight. **(B)** Alanine aminotransferase (ALT), **(C)** aspartate aminotransferase (AST), **(D)** alkaline phosphatase (ALP), **(E)** creatinine and **(F)** blood urea nitrogen (BUN) levels were measured after 2 weeks of drugs treatment. Osi, osimertinib. All data are shown as mean ± SD.

To explore the inhibitory effect of FYLM on tumor progression *in vivo*, we first constructed a xenograft mouse model harboring EGFR-Del19/T790M/C797S-mutant *via* subcutaneously inoculating LLC-triple mutant cells into C57BL/6J mice. Tumor-bearing mice were continuously orally gavage for 2 weeks with 0.5% CMC-Na, FYLM (24 g/kg/d), FYLM (48 g/kg/d), osimertinib (50 g/kg/d), a combination treatment of osimertinib (50 g/kg/d) and FYLM (24 g/kg/d), or a combination treatment of osimertinib (50 g/kg/d) and FYLM (48 g/kg/d). As a result, the body weight of mice in each group steadily increased without significant differences, and no obvious toxicity was observed compared to the control treatment group (Figures 3B, G). The tumor weight and tumor volume of the empty vector group were slightly smaller than those of the overexpression group, but the differences were not statistically significant (Figures 3C–E). FYLM or osimertinib inhibited tumor progression compared with the control group. Among them, the combination of FYLM (48 g/kg/d) and osimertinib had the most significant inhibitory effect (Figures 3H–J). Furthermore, to evaluate the safety of combination treatment with FYLM and osimertinib, we measured spleen weight and biochemical analysis of liver function [alanine aminotransferase (ALT), aspartate aminotransferase (AST), alkaline phosphatase (ALP)] and kidney function [creatinine, blood urea nitrogen (BUN)]. The results showed no significant differences among the groups. Therefore, the combination of FYLM and osimertinib has no significant toxicity (Figures 4A–F).

### 3.4 The combination of FYLM and osimertinib inhibits cell proliferation, regulates cell cycle and promotes apoptosis in triple-mutant EGFR xenograft mouse model

**FIGURE 5:**
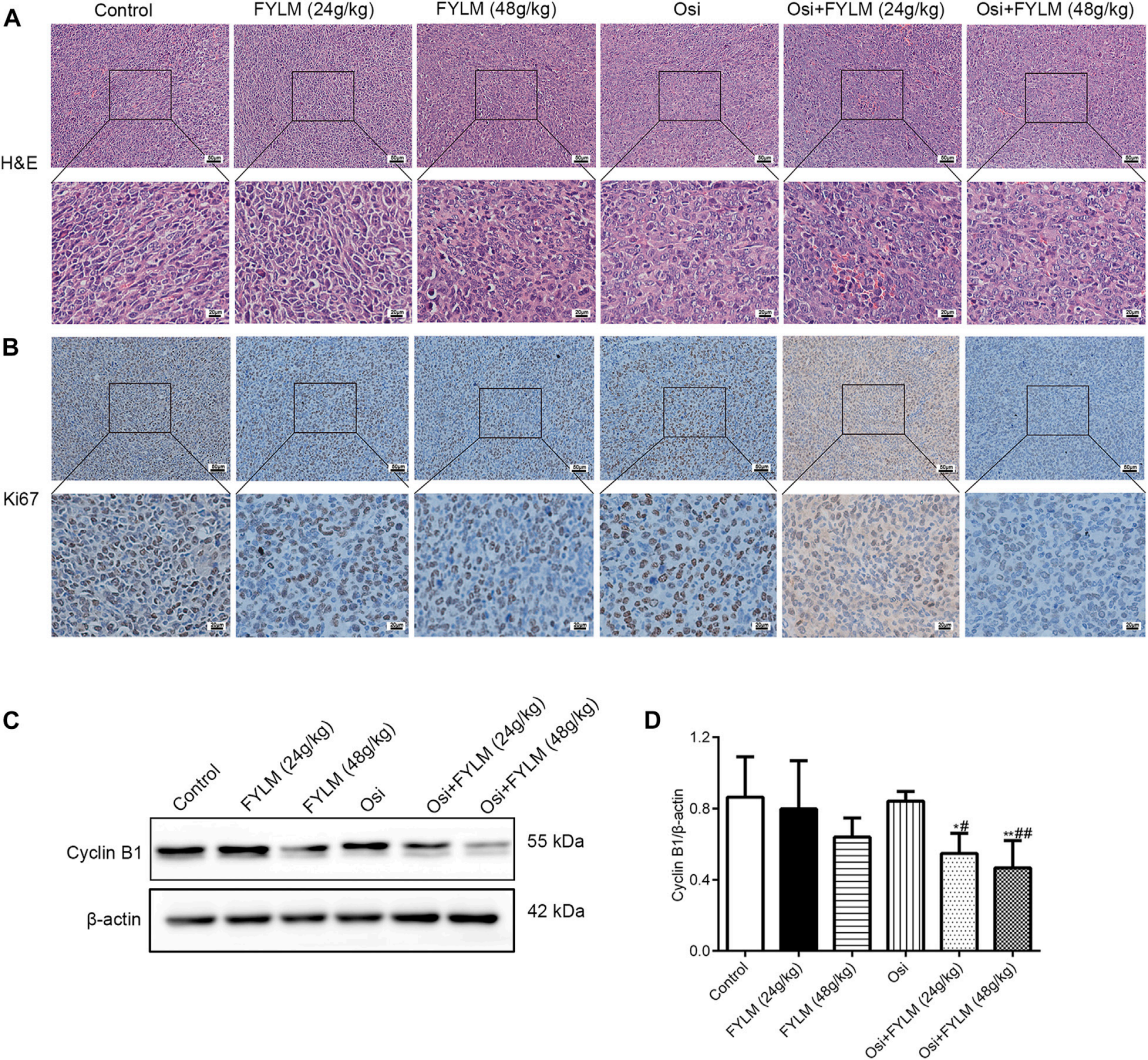
FYLM combined with osimertinib regulates cell proliferation and cell cycle in EGFR-Del19/T790M/C797S LLC tumor-bearing mice. **(A)** HE staining of tumor tissues. **(B)** Detection of Ki67 expression in tumor tissues by immunohistochemistry. **(C)** Cyclin B1 proteins were detected by western blotting. **(D)** Quantitative analysis of Cyclin B1 protein levels. *p < 0.05 and **p < 0.01 vs. Control; ^#^ *p* < 0.05 and ^##^ *p* < 0.01 vs. Osi. Osi, osimertinib. All data are shown as mean ± SD.

**FIGURE 6:**
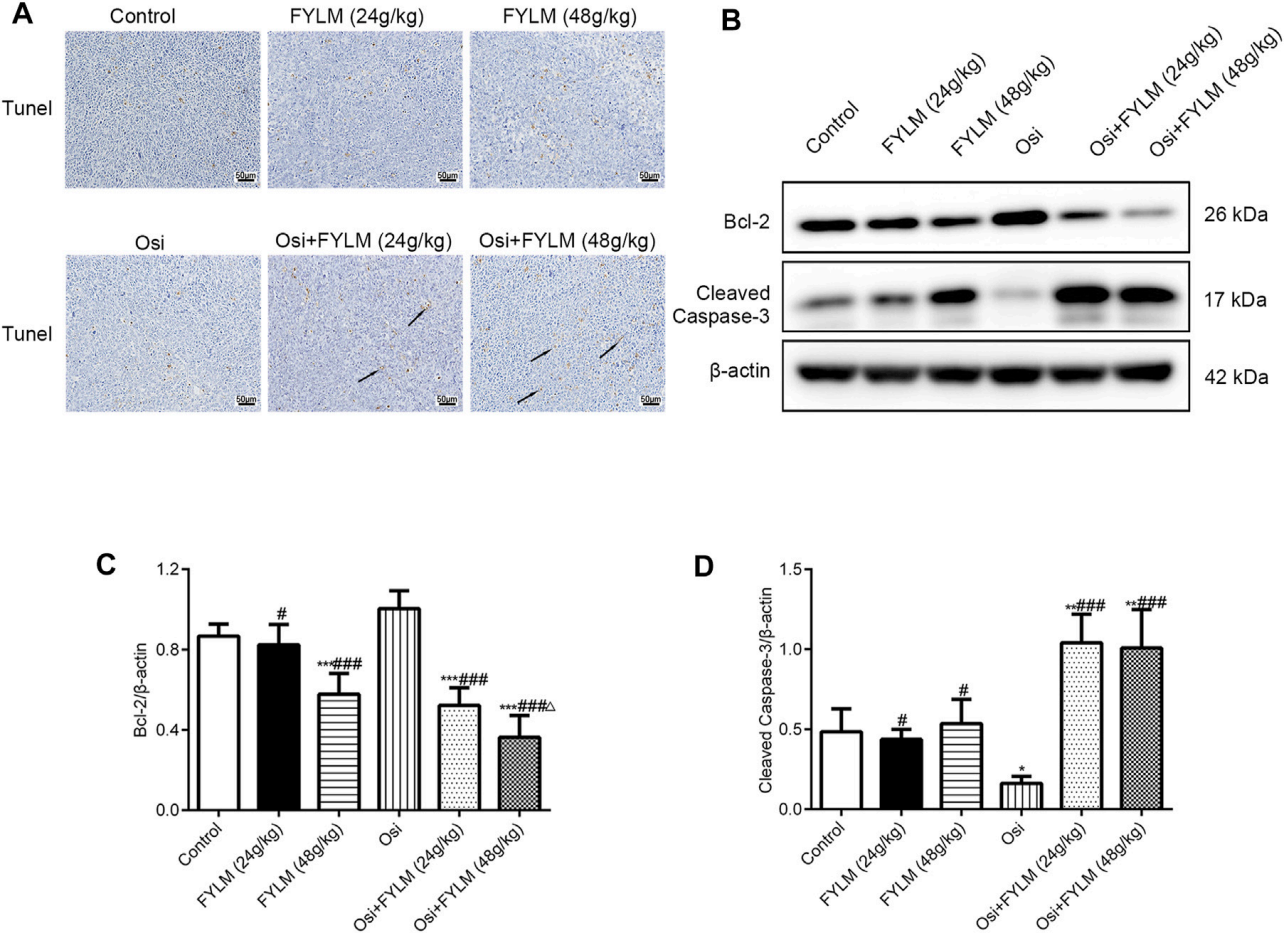
FYLM combined with osimertinib regulates apoptosis in EGFR-Del19/T790M/C797S LLC tumor-bearing mice. **(A)** Detection of apoptosis in tumor tissues by TUNEL staining. Arrows indicate brown-yellow apoptotic cells. **(B)** Bcl-2 and cleaved Caspase-3 proteins were detected by western blotting. **(C,D)** Quantitative analysis of Bcl-2 and cleaved Caspase-3 protein levels. *p < 0.05, **p < 0.01 and ***p < 0.001 vs. Control; ^#^ *p* < 0.05 and ^###^ *p* < 0.001 vs. Osi; ^△^ *p* < 0.05 vs. Osi + FYLM (24 g/kg). Osi, osimertinib. All data are shown as mean ± SD.

HE staining revealed a decrease in nuclear density, lighter staining and lower nucleoplasm ratio in the combined treatment group (Figure 5A). To investigate the effect of FYLM on tumor growth, we detected the levels of cell proliferation, cell cycle, and apoptosis-related protein Ki-67, cyclin B1, Bcl-2 and cleaved Caspase-3 in xenograft mouse tumor tissues by western blot and immunohistochemistry. Consistent with the results of phenotypic experiments, the combination of FYLM and osimertinib reduced the positive expression of Ki-67 protein in a dose-dependent manner in the immunohistochemistry assay (Figure 5B). The western blot results showed that the combination of FYLM and osimertinib downregulated the protein expression level of cyclin B1 (Figures 5C, D). Furthermore, TUNEL staining results illustrated that combination of osimertinib and FYLM (24 and 48 g/kg) increased apoptosis (Figure 6A). Moreover, western blot assays revealed that combination treatment of osimertinib and FYLM (24 and 48 g/kg) inhibited Bcl-2 expression compared with osimertinib or FYLM alone. However, cleaved Caspase-3 protein expression were upregulated in combination treatment of osimertinib and FYLM (24 g/kg) group and the combination treatment of osimertinib and FYLM (48 g/kg) group compared with the osimertinib alone group (Figures 6B–D).

### 3.5 The combination of FYLM and osimertinib inhibits PRC1/Wnt/EGFR pathway in triple-mutant EGFR xenograft mouse model

**FIGURE 7:**
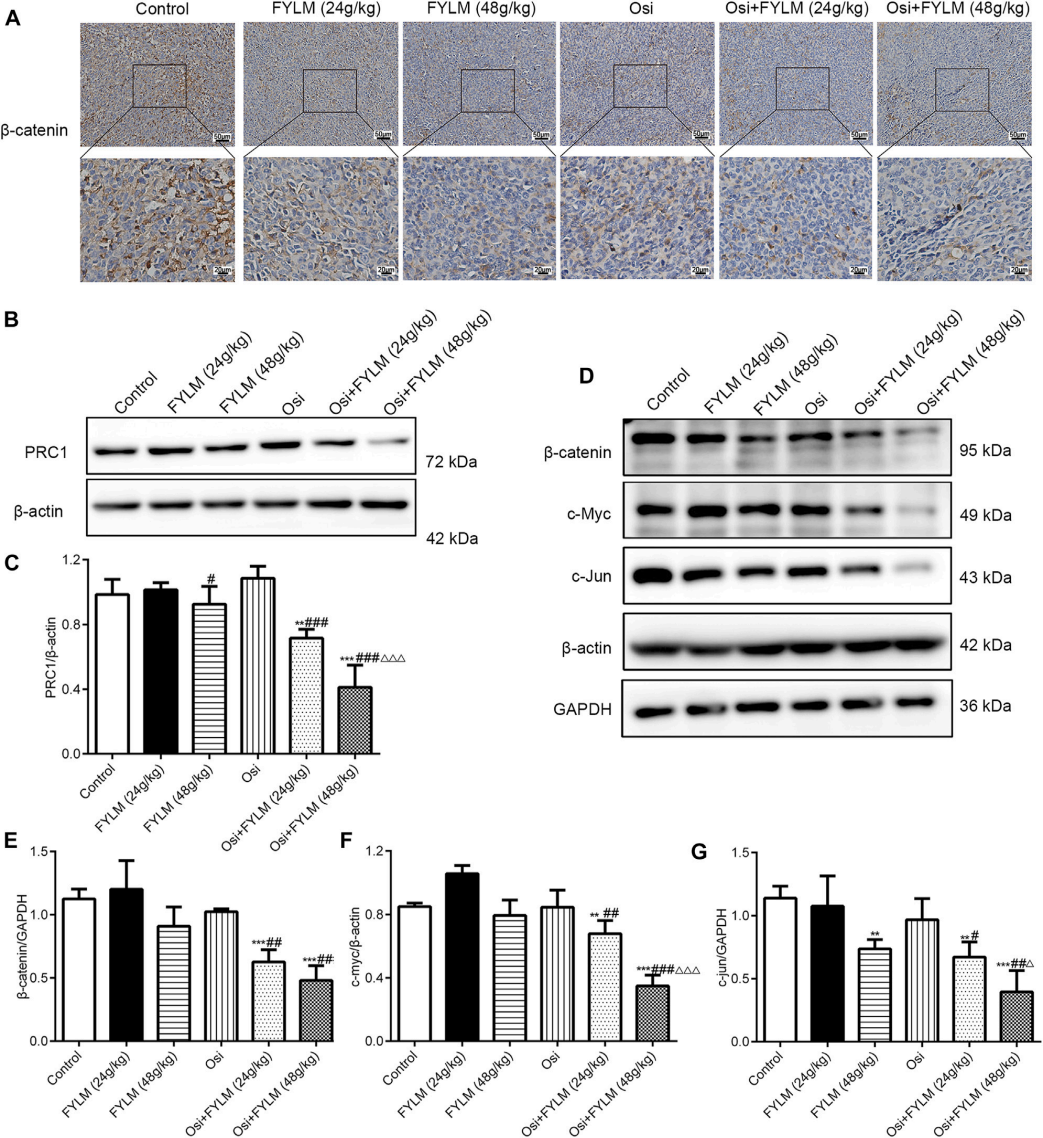
FYLM combined with osimertinib regulated PRC1/Wnt pathway in EGFR-Del19/T790M/C797S LLC tumor-bearing mice. **(A)** Detection of β-catenin expression in tumor tissues by immunohistochemistry. **(B,D)** PRC1, β-catenin, c-Myc and c-Jun proteins were detected by western blotting. **(C,E–G)** Quantitative analysis of PRC1, β-catenin, c-Myc and c-Jun protein levels. **p < 0.01 and ***p < 0.001 vs. Control; ^#^ *p* < 0.05, ^##^ *p* < 0.01 and ^###^ *p* < 0.001 vs. Osi; ^△^ *p* < 0.05 and ^△△△^ *p* < 0.001 vs. Osi + FYLM (24 g/kg). Osi, osimertinib. All data are shown as mean ± SD.

**FIGURE 8:**
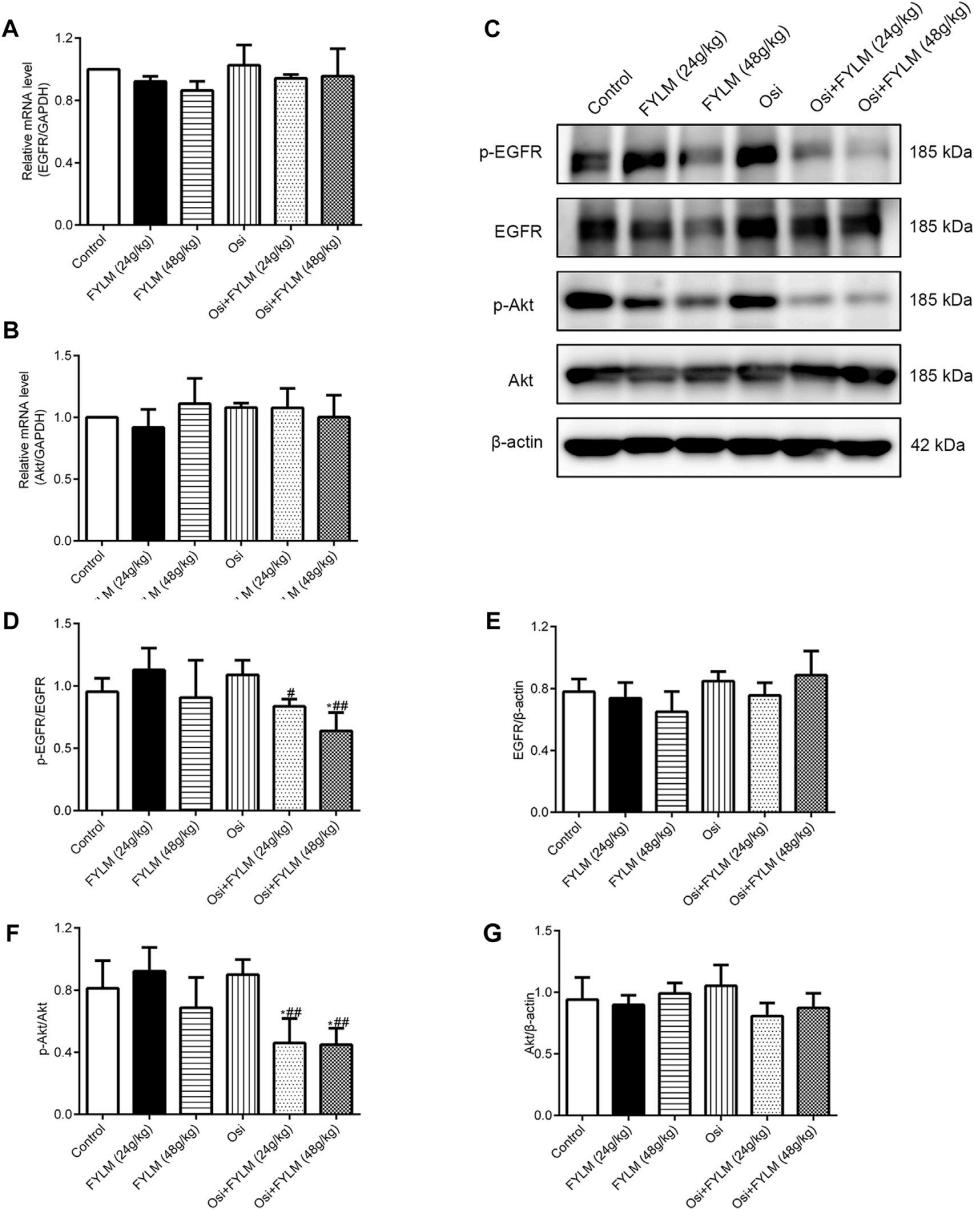
FYLM combined with osimertinib regulated EGFR pathway in EGFR-Del19/T790M/C797S LLC tumor-bearing mice. **(A,B)** mRNA levels of EGFR and Akt detected by real-time qPCR. **(C)** p-EGFR, EGFR, p-Akt and Akt proteins were detected by western blotting. **(D–G)** Quantitative analysis of p-EGFR/EGFR, EGFR, p-Akt/Akt and Akt protein levels. *p < 0.05 vs. Control; ^#^ *p* < 0.05, ^##^ *p* < 0.01 vs. Osi. Osi, osimertinib. All data are shown as mean ± SD.

To clarify the mechanism synergistic anti-cancer effect of FYLM, we investigated the protein expression of PRC1, Wnt pathway-related protein (β-catenin, c-Myc, c-Jun) and EGFR pathway-related protein (p-EGFR, EGFR, p-Akt and Akt) in xenograft tumor tissues by western blot and immunohistochemistry. Immunohistochemistry assays showed a decrease of β-catenin protein in both the combination treatment of osimertinib and FYLM (24 g/kg) group and the combination treatment of osimertinib and FYLM (48 g/kg) group (Figure 7A). Western blot assays revealed that combination treatment of osimertinib and FYLM (24 and 48 g/kg) suppress PRC1, β-catenin, c-Myc and c-Jun expression relative to the treatment with osimertinib or FYLM alone (Figures 7B–G). The Wnt pathway and the EGFR pathway are key pathways related to drug resistance, and they crosstalk each other. Inhibition of Wnt pathway and EGFR pathway can delay drug resistance. No significant changes in EGFR and Akt expression in the combination treatment group compared to other individual treatment groups in terms of real-time qPCR and immunoblotting of mouse tumor tissues (Figures 8A, B, E, G). However, the combination of osimertinib and FYLM (24 and 48 g/kg) reduced the expression of p-EGFR/EGFR and p-Akt/Akt ratios (Figures 8C, D, F). These results suggest that the combination of FYLM and osimertinib inhibits the PRC1/Wnt/EGFR pathway, which may be the mechanism of FYLM alleviating TKI resistance.

## 4 Discussion

Based on the basic treatment method of strengthening healthy qi to eliminate pathogens, FYLM consists of two types of herbs: Huangqi, Baizhu, Renshen and Gancao to replenish healthy qi; and Banzhilian, Baihuasheshecao, Fuling, Zhebeimu, Shancigu, Yiyiren to eliminate unhealthy trends. Studies have shown that during the treatment of lung cancer, whether it is surgery, radiotherapy, chemotherapy or targeted therapy, the healthy qi is obviously damaged, manifested as fatigue, gastrointestinal reactions, rashes and other side effects (Krzyzanowska et al., 2021; Mössner, 2022). In addition, Chinese medicine compounds have shown a multitude of advantages in the treatment of lung cancer, such as synergistic inhibition of tumor growth, sensitivity to targeted therapy, and reduction of toxicity (Li et al., 2016; Shao et al., 2021). Hence, the Chinese medicine compound combined with targeted drugs is a promising treatment option for the treatment of TKI-resistant NSCLC. Clinically, FYLM has a good effect on the treatment of lung cancer and can reduce the adverse reactions of patients. In this study, we analyzed the major components of FYLM using UPLC-Q-Orbitrap-MS and identified 36 chemical constituents. Among them, it has been reported that quercetin, scutellarin, oleanolic acid, apigenin and formononetin can overcome acquired resistance to EGFR-TKIs by inhibiting proliferation, promoting apoptosis and inducing autophagy *in vitro* and *in vivo* (Chen et al., 2019a; Chen et al., 2019b; Yu et al., 2020b; Huang et al., 2021; Sun et al., 2021). For example, quercetin reduced the growth of EGFR-C797S mutated NSCLC cells *via* inhibiting ALX and promoting apoptosis. Similarly, the current study confirmed the role of FYLM in overcoming EGFR-TKI resistance in EGFR-mutated NSCLC. Specifically, we discovered that serum-containing FYLM inhibited cell growth both in triple-mutant cells and T790M-mutant cells. Animal experiments showed that the combination of FYLM and osimertinib suppressed tumor growth in LLC triple-mutant EGFR xenograft mice. In addition, the pathological changes of the tumor were observed by HE staining, and it was found that FYLM improved the malignancy of the tumor tissue in the xenograft model, decreased the density of nuclear, and decreased the nuclear-cytoplasmic ratio. Moreover, immunohistochemical results showed that FYLM also reduced the level of Ki-67 protein, and the effect of FYLM combined with osimertinib was more obvious. Ki-67 is mainly used to mark tumor cells in the proliferative cycle, and elevated Ki67 has a poor prognosis (Uxa et al., 2021). This suggests that the combination of FYLM and osimertinib has a synergistic effect in inhibiting the proliferation of EGFR-mutant NSCLC *in vitro* and *in vivo*.

Interestingly, we investigated the cyclin B1 (a cell cycle regulator) expression in mouse tumor tissues and found that the combination of FYLM and osimertinib downregulated the protein expression level of cyclin B1. Decreased expression of cyclinB1 causes G2 arrest, which is implicated in the mechanism of action of certain anticancer drugs (Lv et al., 2020). A previous report showed that the proliferation inhibition of NSCLC cells by sulforaphane was associated with cell cycle arrest caused by decreased cyclin B1 expression (Żuryń et al., 2019). It is well known that the most common hallmarks of cancer cells include sustained proliferation and attenuated apoptosis (Hanahan and Weinberg, 2011). Lately, research has shown that deoxypodophyllotoxin plays an important role in acquired resistance to gefitinib by inducing apoptosis in NSCLC cells (Kim et al., 2021). Consistent with the results, in our study, FYLM downregulated the expression of the apoptosis-related protein Bcl-2 while upregulating the cleaved caspase-3 in a mouse xenograft model. Furthermore, we investigated ALT, AST, ALP, CRE and BUN levels in the serum of xenograft-bearing mice and demonstrated that FYLM had no significant hepato-nephrotoxic side effects. In short, considering safety and efficacy, FYLM may be a natural, safe and promising adjunctive drug for the treatment of NSCLC harboring EGFR mutations. Overall, the above results suggest that FYLM reduces proliferation and arrests the cell cycle while increasing apoptosis to delay osimertinib resistance.

EGFR is a transmembrane receptor with tyrosine kinase activity that activates downstream signaling pathways *via* ligand-mediated autophosphorylation of intracellular tyrosine kinase domains (da Cunha Santos et al., 2011). Typically, EGFR downstream pathways are implicated in cell proliferation signaling, apoptosis, invasion, metastasis and angiogenesis (Levantini et al., 2022). Somatic mutations in the EGFR gene lead to ligand-independent activation of EGFR growth factor signaling (Harrison et al., 2020). Osimertinib, a potent EGFR inhibitor, competitively binds to the ATP site of the tyrosine kinase domain of EGFR, inhibits EGFR autophosphorylation, blocks the cell cycle and promotes apoptosis of tumor cells (Leonetti et al., 2019). Unfortunately, resistance mutations reduce osimertinib binding to EGFR, i.e., the development of acquired resistance, marking the agent’s less effective inhibition of this pathway (Du et al., 2021). To date, no drug has been used to treat the EGFR^Del19/T790M/C797S^ mutation caused by osimertinib. In our research, we chose a mouse Lewis lung carcinoma cell line harboring the Del19/T790M/C797S mutation generated by lentiviral transfection as a model of osimertinib resistance. Moreover, the H1975 cell line carrying the L858R/T790M mutation as control cells was sensitive to osimertinib. The triple mutant LLC cells were then injected subcutaneously into C57BL/6 mice to create an animal xenograft model. In addition, mouse Lewis lung cancer has been widely used as an experimental model for tumor research, especially in anti-tumor drug screening (Xu et al., 2018; Wang et al., 2021a). In *in vitro* study, we demonstrated that serum-containing FYLM downregulated the expression of p-EGFR protein in a dose-dependent manner, and the effect of the combination with osimertinib and FYLM was more obvious in triple-mutant cells. Similarly, FYLM synergizes with osimertinib to reduce the protein levels of p-EGFR and p-Akt in LLC triple-mutant tumor-bearing mice. The data show that combined treatment with FYLM and osimertinib inhibits the phosphorylation of EGFR in drug-resistant cells and triple-mutant xenografts.

Moreover, studies have shown that PRC1 is associated with poor prognosis in lung adenocarcinoma, and thus inhibition of PRC1 may be a promising therapeutic target for lung adenocarcinoma (Chen et al., 2016). More importantly, gene silencing of PRC1 could reduce the expression of β-catenin, cyclin D2, c-Myc and c-Jun in Wnt/β-catenin pathway in NSCLC cell lines (Zhan et al., 2017). In addition, the Wnt/β-catenin pathway mainly regulates important cellular functions such as cell proliferation, differentiation and apoptosis, and is involved in tumorigenesis and drug resistance of NSCLC (Stewart, 2014). In erlotinib-resistant HCC827/ER cells, suppressing the activation of the Wnt/β-catenin signaling can inhibit erlotinib resistance and cell migration (Wang et al., 2020). At the same time, EGFR mutations lead to nuclear accumulation of β-catenin, which activates the conduction of the Wnt pathway. The Wnt signaling pathway interacts with the EGFR signaling pathway to jointly regulate EGFR-TKI resistance. In our study, we discovered that FYLM not only decreased the PRC1 protein levels but also β-catenin, c-Myc and c-Jun protein levels in xenograft models. All of these results suggest that FYLM is associated with downregulation of PRC1 and Wnt/β-catenin pathway expression, reduced proliferation and increased apoptosis, thereby delaying resistance to osimertinib in drug-resistant cells and triple-mutant xenografts. Notably, FYLM can sensitize resistance-mutant NSCLC to osimertinib by affecting the PRC1/Wnt/EGFR pathway. However, this pathway may be regulated by multiple factors, and our current research did not carry out a retrospective experiment. In the next phase of research, pathway inhibitors should be applied for further validation. Another limitation of this study is that we only evaluated the safety of liver function and kidney function in tumor-bearing mice, and further toxicity tests of FYLM still need to be conducted.

## 5 Conclusion

**FIGURE 9:**
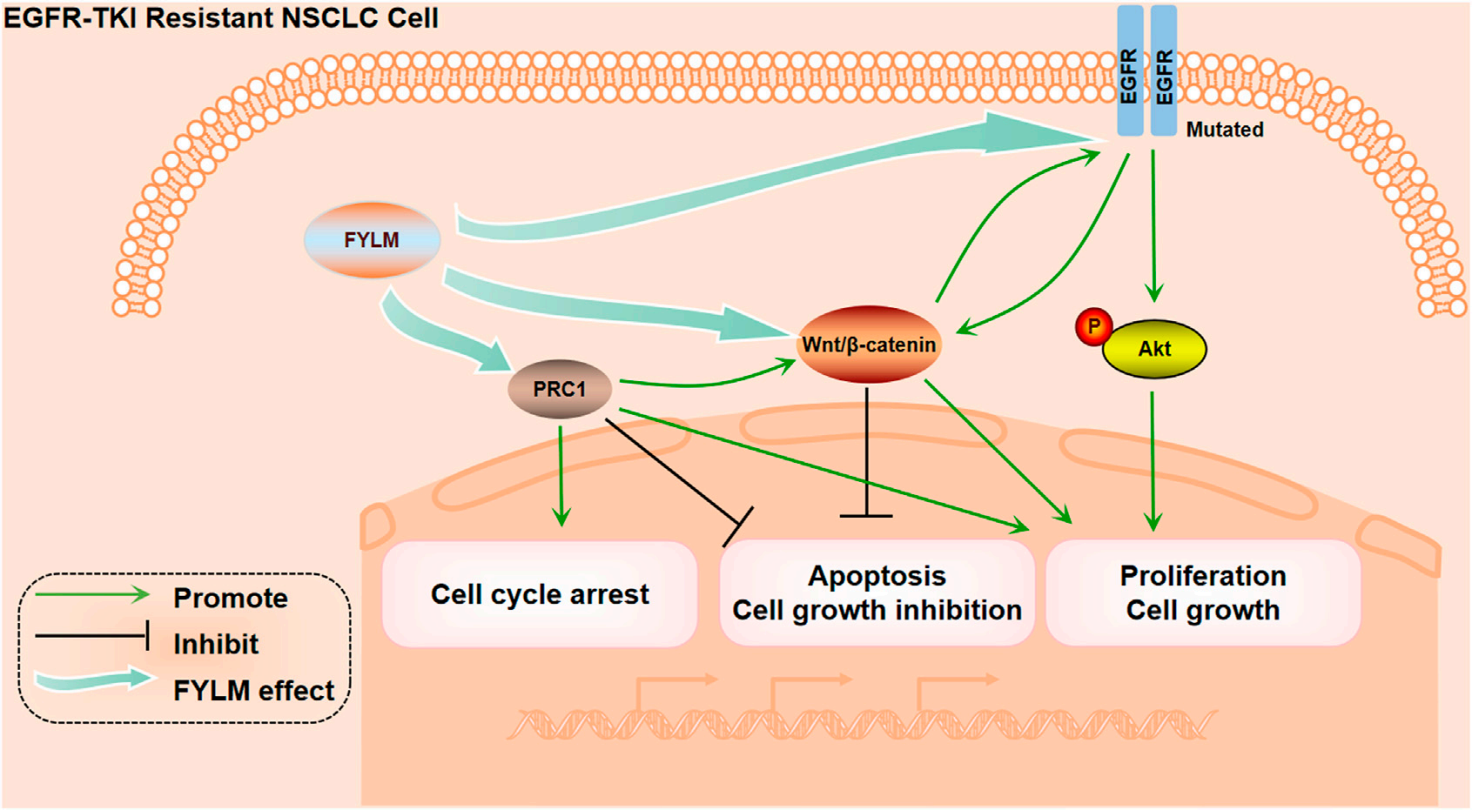
The Mechanism diagram of FYLM alleviating drug resistance.

In summary, our study confirmed that FYLM synergistically reduces proliferation and increases apoptosis in EGFR mutant NSCLC cells. The mechanism of FYLM alleviating drug resistance may be related to reducing the expression of PRC1 and reducing the expression of Wnt pathway-related proteins. Furthermore, FYLM may be a promising adjunctive drug for patients with EGFR-mutant advanced NSCLC (Figure 9). The disadvantage of this study is that it only studied the research of FYLM in the treatment of lung cancer, and did not involve the research of FYLM in the treatment of other types of cancer. In order to better develop the medicinal value of FYLM, further research needs to be done.

## Data Availability

The original contributions presented in the study are included in the article/Supplementary Material, further inquiries can be directed to the corresponding author.

## Abbreviations

NSCLC, non-small cell lung cancer; EGFR, epidermal growth factor receptor; Del19, exon 19 base deletions; L858R, point mutation in exon 21; NCCN, National Comprehensive Cancer Network, TKIs, tyrosine kinase inhibitors; EMT, epithelial-mesenchymal transition; PRC1, protein regulator of cytokinesis 1; TCM, Traditional Chinese Medicine; FYLM, Feiyiliu Mixture; LLC, Lewis lung carcinoma; MOI, multiplicity of infection; ALT, alanine aminotransferase; AST, aspartate aminotransferase; ALP, alkaline phosphatase; CRE, creatinine; BUN, blood urea nitrogen.
